# Plasma IgM Levels Differentiate between Survivors and Non-Survivors of Culture-Positive and Culture-Negative Sepsis and SIRS: A Pilot Study

**DOI:** 10.3390/jcm10225391

**Published:** 2021-11-19

**Authors:** Navichandra Pathare, Tamas Szakmany, Judith E. Hall, Meike Heurich

**Affiliations:** 1 School of Medicine, College of Biomedical and Life Sciences, Cardiff University, Cardiff CF14 4YS, UK; navinpathare@gmail.com; 2 Department of Anaesthesia, Intensive Care and Pain Medicine, Division of Population Medicine, Cardiff University, Cardiff CF14 4XN, UK; hallje@cardiff.ac.uk; 3 ACT Directorate, Royal Glamorgan Hospital, Llantrisant CF72 8XR, UK; 4 Critical Care Directorate, Grange University Hospital, Cwmbran NP44 2XJ, UK; 5 Faculty of Health Sciences and Veterinary Medicine, University of Namibia, Windhoek 13301, Namibia; 6 School of Pharmacy and Pharmaceutical Sciences, College of Biomedical and Life Sciences, Cardiff University, Cardiff CF10 3NB, UK

**Keywords:** immunoglobulins, sepsis, SIRS, culture-negative

## Abstract

Immunoglobulin IgM is important for controlling viral and bacterial infections, and low immunoglobulin levels have been found in sepsis. There is a clear need to stratify sepsis patients according to the presence of an invading organism, compared to no organism identified, and SIRS patients, where organ dysfunction is a result of a non-infective process. The aim of this pilot study in a small cohort of patients with sepsis was to evaluate the association between IgM plasma levels and survival in 47 patients with sepsis and 11 patients diagnosed with organ failure without the identification of a pathogen (SIRS). Patients were admitted to the intensive care unit (ICU) at The Royal Glamorgan Hospital, Llantrisant, UK between 2010 and 2014. We found that low IgM levels were associated with sepsis, but not SIRS. IgM levels did not differ significantly for culture-positive (CP) compared with culture-negative (CN, no organism found) sepsis samples. Kaplan–Meier analysis was used to compare survival curves according to IgM levels, with no significant difference. We observed significantly higher survival in the CP samples when comparing with CN. Cut-off value for IgM (266 μg/mL) for diagnosis of sepsis patients was determined using receiver operator characteristic (ROC) curves with 70% sensitivity, 69% specificity and 92% negative predictive values (NPV), respectively. The corresponding area under the curve (AUC) for the discrimination of sepsis patients was AUC = 0.73, and in a subgroup analysis of CP was AUC = 0.77 and for CN was AUC = 0.79. We confirm IgM as a good diagnostic marker of sepsis. These findings indicate a difference in the pathology between culture-positive versus negative sepsis, SIRS and survival. This indicates that IgM is likely relevant to pathology, because of its role in the early immune response against pathogens, the potentially protective role of natural IgM antibodies, and supports its application in immunoglobulin therapy.

## 1. Introduction

Sepsis is defined as the dysregulated host response to infection causing organ dysfunction [1]. This recent definition closely mirrors the previous category of severe sepsis, which is a major cause of morbidity and mortality in both developed and developing countries [2]. Mortality rates remain at around 30%, and higher in septic shock, despite advances in critical care [3]. The invasion of sterile tissues by infective agents will trigger a primarily innate immune response, which could lead to the clinical manifestation of sepsis and severe sepsis pathology [4]. Initially, it was assumed that this was primarily due to Gram-negative bacteria, but it is now clear that Gram-positive bacteria, as well as viral, fungal and parasitic organisms, also play an important role in the development of sepsis [5,6,7,8]. A retrospective, longitudinal study over a 20-year period reported that in over 50% of sepsis patients, microbiologically proven culture-positive (CP) samples were recorded [9] The invading organism distribution showed Gram-positive bacteria (52.1% of cases), Gram-negative bacteria (37.6%), polymicrobial infections (4.7%), anaerobes (1.0%), and fungi (4.6%) [9]. The organism class responsible for the primary infection, has been shown to play a role in determining the mortality of patients with sepsis. We have previously demonstrated that primarily Gram-negative infections are associated with an elevated mortality [8]. However, often no specific organism can be identified, and 28–49% of severe sepsis incidents have been described as being culture-negative (CN) [10,11]. This is commonly explained by a lack of test sensitivity for infecting organisms due to insensitive methodologies applied in the clinical practice or administration of antibiotics [12], but could also be a result of pathophysiological differences between culture-positive and negative sepsis or SIRS [13].

Low immunoglobulin levels have been found in sepsis [14,15,16,17,18]. The IgM isotype is produced by B cells in responses to acute infection, thus endogenous IgM is the first line of the humoral host defense to aid opsonization and clearance of invading organisms [19,20,21]. IgM has been shown to be crucial for controlling both viral and bacterial infections [22,23,24], as its absence leads to inefficient induction of protective IgG antibody responses [25,26]. Low IgM levels have been shown to be associated with sepsis [27,28,29], possibly caused by a defective B cell response or a selective depletion of IgM producing memory cells [30,31], which may affect early pathogen clearance. Some evidence indicates that IgM-enriched therapy may be beneficial in Gram-negative sepsis, however the data is conflicting [32,33,34]. Unknown mechanisms of action of both endogenous immunoglobulins and immunoglobulin preparations in sepsis could explain the controversial results found in clinical trials [35].

There is a clear need to stratify cohorts of patients with clinical manifestation of sepsis into populations according to presence of an invading organism (culture-positive, CP) and its Gram status (Gram-positive versus Gram-negative), compared to absence of an invading organism (culture-negative, CN, i.e., no organism identified) and compare them to patients where organ dysfunction is a result of a non-infective process known as systemic inflammatory response syndrome (SIRS) [13]. There is an ongoing need to identify biomarkers that aid this stratification, as they might reveal important subgroups of patients needing targeted therapeutics and treatment [4]. Therefore, the aim of our study was to compare the endogenous plasma IgM levels in culture-positive (including Gram-positive and negative) to culture-negative (no organism found) severe sepsis and SIRS patients and examine these levels in survivors and non-survivors.

## 2. Materials and Methods

### 2.1. Study Design

Following written consent, patients presenting to the Royal Glamorgan Hospital’s 10-bedded mixed medical/surgical ICU between January 2011 and March 2014 were enrolled. The study was approved by the South East Wales Research Ethics Committee (reference number 10WSE/421, June 2011) and registered with the UK Clinical Research Network (UKCRN; Cellular and biochemical investigations in sepsis, ID 11231).

#### 2.1.1. Inclusion Criteria

We recruited adult patients, 18 years or older, who presented to the ICU within 24 h of the presumed onset of the acute illness. The diagnosis of sepsis required the presence of systemic inflammatory response syndrome and organ dysfunction due to infection according to the previous definition of sepsis and had to fulfil the following criteria [2]:

(a) presence of at least 2 out of 4 SIRS criteria: (i) Temperature >38 °C or <36 °C. (ii) Heart rate > 90 beats per minute. (iii) Respiratory rate > 20/min or PaCO_2_ < 32 mmHg or need for mechanical ventilation. (iv) White cell count > 12,000 or <4000 cells/+ or >10% band forms in whole blood. (b) Known or suspected bacterial infection requiring antimicrobial therapy. (c) Organ dysfunction (one of the following): (i) Circulatory (one of the following): Systolic blood pressure < 90 mmHg or mean arterial pressure < 70 mmHg for 1 h despite adequate volume replacement. (ii) Respiratory (one of the following): PaO_2_/FiO_2_ ratio < 250 mmHg in the presence of other organ failure. PaO_2_:FiO_2_ ratio < 200 mmHg in primary pulmonary failure. (iii) Renal: Urine output < 0.5 mL/kg/h for 1 h despite adequate volume replacement. (iv) Haematological: Platelet count < 80,000 cells per mm^3^. (v) Metabolic (one of the following): Unexplained acidosis with pH < 7.3 or BE < −5, or Lactate > 1.5 normal upper limit for laboratory.

Patients in the SIRS group were enrolled when they fulfilled two or more SIRS criteria, had documented organ dysfunction as described above, but were not treated with antimicrobials for known or presumed infection. All patients had arterial cannulation as part of their standard care. Further clinical details of the groups have been described before [8,36].

#### 2.1.2. Exclusion Criteria

We only recorded the first ICU admission and excluded readmissions. We excluded currently pregnant, breastfeeding, or females in whom a pregnancy test had not been performed. Patients unlikely to survive for the duration of the study period or patients who suffered a cardiac arrest before admission to the ICU were excluded. We excluded patients with underlying impairment of higher function that would have made it impossible for informed consent to be given upon recovery (e.g., severe learning disability). Patients with severe immune deficiency including AIDS diagnosis, those on immunosuppressant drugs or high dose corticosteroid treatment (>10 mg prednisolone equivalent per day) as well as liver failure with Child–Pugh grade 3 or greater were excluded.

#### 2.1.3. Healthy Controls

Healthy controls were normally fit and well; not suffering from an acute or chronic inflammatory illness (e.g., severe asthma or rheumatoid arthritis) and not taking immunosuppressant medications

### 2.2. Blood Plasma Collection and IgM Quantification

Blood was drawn from consenting healthy volunteers (*n* = 48) and sepsis (*n* = 47) or SIRS (*n* = 11) patients within 12 h of ICU admission and plasma was obtained by centrifugation from whole blood with added EDTA and stored in 0.5 mL aliquots at −80 °C.

IgM protein levels were determined by ELISA developed in house. In brief, 96 well micro titer plates were coated with anti-IgM capture antibody (donkey Anti-Human IgM, Fc5µ fragment specific) at 5 µg/mL (Stratech Scientific Limited, Ely, UK) in 50 mM Carbonate buffer, pH 9.6 for 2 h at 37 °C, washed once with 5% PBST, followed by blocking with 5% gelatin in PBST for 1 h at 37 °C. Cross-reactivity was negligible according to the manufacturer: based on immunoelectrophoresis and/or ELISA, the antibody reacts with the Fc5µ portion of the human IgM heavy chain, but not with human IgG, IgA, or the light chains of human immunoglobulins.

Patient or healthy control plasma samples were measured in triplicate (*n* = 3) and diluted 1:2000 in 5% gelatin/PBST and incubated for 1 h at 37 °C, followed by PBST wash. Goat anti-human IgM-HRP (1:5000 dilution) was incubated for 1 h at 37 °C, and washed thrice with 5% PBST. O-Phenylenediamine dihydrochloride (OPD) (Sigma Aldrich Co Ltd., UK) is a horse radish peroxidase (HRP) substrate that was added to each well for 10 min before the reaction was stopped with 10% sulphuric acid. Absorbance was read at optical density (OD) of 492 nm in a plate reader (Tecan Ltd., UK). Endogenous plasma IgM concentration was calculated by linear regression analysis against a IgM standard (Sigma Aldrich Co Ltd., UK). The IgM ELISA was validated by comparison to the clinical standard, IgM nephelometry (Department of Clinical Biochemistry, University Hospital Wales, Cardiff, UK) with a correlation of r = 0.86 (Spearman r) and *p* = 0.001.

The IgM ELISA normal range was determined in healthy plasma samples as 92–1160 μg/mL, with sensitivity of 5–5000 μg/mL. IgM was determined as intra-and inter-assay triplicates. To determine the cut-off IgM concentration, Youden’s J index was determined from the ROC curve of the sepsis cohort. The cut-off value of 266 μg/mL showed 70% sensitivity and 69% specificity. With a cut-off value of <266 μg/mL, IgM was considered a good diagnostic marker to distinguish between sepsis and healthy controls or SIRS.

As marker of inflammation and discriminator between sepsis and SIRS, the standard clinical marker C-reactive protein (CRP) (day 1) was determined by the hospital central laboratory using a immunoturbidometry assay (Roche Diagnostics, USA) according to manufacturer’s instructions. The assay reports a range of 0.3–350 mg/L (Limit of detection, LoD = 0.3 mg/L). TNF-α, IL-1β, IL-6, and IL-8 were determined utilizing the human proinflammatory 9-plex kit (Meso Scale Discovery (MSD), Meso Scale Diagnostics, Rockville, USA).

The Sequential Organ Failure Assessment (SOFA) score calculates the number and the severity of organ dysfunction in six organ systems (respiratory, coagulation, liver, cardiovascular, renal, and neurologic) to predict ICU mortality [37,38].

### 2.3. Microbiology

The main organism causing sepsis was defined as the presence of a positive bacterial or viral identification in any sample taken from 72 h preceding recruitment to 72 h following admission to the ICU captured using the hospital’s electronic microbiology patient database. The microbiological techniques used in the study hospital for diagnostics included standard microscopy and culture, viral PCR studies, and urine *Legionella* antigen testing. Advanced bacterial identification systems such as PCR or Matrix Assisted Laser Desorption/Ionization (MALDI-TOF) based techniques were not used.

One independent intensive care doctor and one independent microbiologist were consulted to confirm that any given positive result would be appropriate when considering the clinical context and other supporting clinical results. This was adjudicated with full access to patients’ clinical notes, electronic clinical results and a discussion with the clinical consultant intensivist responsible for the patients’ care. Culture results considered contaminations were excluded from further analysis. Patients with no relevant positive results were classed as culture-negative. Infections were then grouped into Gram-positive and Gram-negative species.

### 2.4. Statistical Analysis

Data was tested for normal distribution according to D’Agostino and Pearson. Mann–Whitney U test was used to analyze not normally distributed data and unequal variances *t*-test for normally distributed data of unequal sample size, as appropriate. We assessed correlations between parameters using the Spearman Rho’s correlation test. A result was considered to be significant at *p* < 0.05. To determine survival from data time points, we generated Kaplan–Meier curves. To determine sensitivity and specificity of the IgM test, receiver operating characteristic (ROC) curves were plotted and the respective areas under the curve were calculated. Youden’s J index was independently calculated using Microsoft Excel 2010 to determine the maximum J index to select the optimum cut-off point for the diagnostic test. All analyses were performed using GraphPad prism8.00 for Windows (GraphPad Software, La Jolla, CA, USA).

## 3. Results

We recruited 47 patients with sepsis and 11 patients with non-infective origin of organ dysfunction (SIRS) during the study period. All patients in the sepsis group were treated with broad-spectrum antibiotics according to local antimicrobial policy and microbiology advice whilst none of the SIRS patients were treated with antimicrobials on ICU admission. Piperacillin/tazobactam, gentamicin, metronidazole, clarithromycin and meropenem was used in the majority of cases either as a monotherapy or a combination. Baseline characteristics of the patients are presented in Table 1.

Whereas the SIRS cohort differed significantly in age and sex distribution, we could see no effect on ICU or hospital days nor APACHE II score. SOFA score appeared to be lower in the SIRS cohort at day 1 and particularly at day 5. IgM levels were significantly reduced in the sepsis, but not in the SIRS cohort.

Microbiological findings included the identification of Klebsiella oxytoca, Staphylococcus aureus, Staphylococcus epidermidis, Streptococcus pneumoniae, Escherichia coli, Enterobacter asburiae, Enterobacter cloacae, Enterococcus faecalis, Pseudomonas aeruginosa, Haemophilus influenzae. Two patients had positive PCR for Influenza A virus, both of them had also Streptococcus pneumoniae infection.

While we observed a statistically significant difference for IgM levels between sepsis and SIRS overall (*p* = 0.03), no significant difference in IgM levels was determined between sepsis survivors and non-survivors (*p* = 0.3132), and could not be established for SIRS patients due to low numbers.

### 3.1. Comparison of Plasma Immunoglobulin M (IgM) Levels in Sepsis, SIRS and Healthy Controls

**Figure 1:**
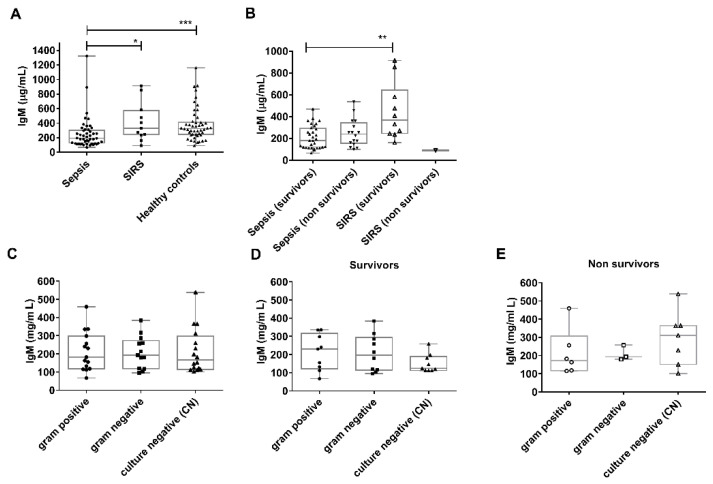
IgM levels in sepsis and SIRS groups. (**A**) IgM levels are decreased in sepsis compared with SIRS (*p* = 0.03) and with healthy controls (*p* < 0.0001). (**B**) IgM levels are not significantly different in sepsis survivors compared to non-survivors (*p* = 0.31), but differed for sepsis survivors compared with SIRS survivors (*p* = 0.002). (**C**) IgM levels are not significantly different in gram positive, negative or culture-negative sepsis (*p* = 0.31), nor for (**D**) survivor, or (**E**) non-survivors. Boxes represent medians and inter quartile ranges, whiskers represent range. Individual values are overlayed on the plots. P values * *p* < 0.05, ** *p* < 0.01, *** *p* < 0.001 were determined by one-way ANOVA and Mann Whitney test. SIRS: Systemic inflammatory response syndrome.

IgM levels in sepsis patients were significantly decreased compared to SIRS (*p* = 0.03) and healthy controls (*p* < 0.0001) with a median and interquartile range of 193 μg/mL (120–310) for sepsis patients (*n* = 47), 331 μg/mL (236–581) for SIRS patients (*n* = 11), and 326 μg/mL (238–420) for healthy controls (*n* = 48) (Figure 1A). There was no statistically significant difference in IgM between sepsis survivors and non-survivors (*p* = 0.31) but sepsis survivors compared with SIRS survivors (*p* = 0.002) (Figure 1B and Table 1).

### 3.2. Comparison of Plasma Immunoglobulin M (IgM) Levels in Severe Sepsis According to Gram-Status

The Gram status could be determined in 28 patients, with 15 identified with a Gram-positive and 13 with a Gram-negative infection. 16 patients were CN (no infectious pathogen identified). The characteristics of 28 CP (combined Gram-positive and negative) patients and 16 CN patients are described in Table 2.

We observed no age difference when comparing CP and CN patients. Gender was unevenly distributed, with more females in the CN cohort. A significant difference in ICU length of stay (LOS) was observed, with shorter ICU LOS in the CN sample cohort (*p* = 0.02). However, hospital LOS was not significantly different between the two groups. There were differences in IgM levels when we compared CP and CN sepsis survivors and non-survivors. However, this remained a statistically non-significant trend, as a result of data distribution. Further, CRP was further reduced in the CN cohort. Patients with positive microbiology (CP) had longer ICU stay, with no difference in admission APACHE II or SOFA scores.

**Figure 2:**
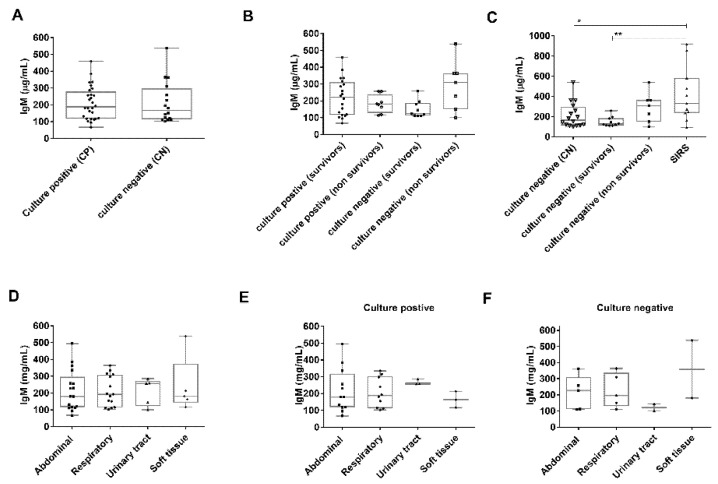
IgM levels in the sepsis groups. (**A**) IgM levels are not significantly different in culture-positive compared with culture-negative sepsis (*p* = 0.78), nor for (**B**) culture-positive or negative survivors compared with non-survivors. (**C**) IgM levels were significantly different comparing culture-negative sepsis and SIRS (*p* = 0.025) and culture-negative sepsis survivors and SIRS (*p* = 0.0055). No significant differences in IgM levels were observed when comparing sepsis (**D**) site of infection, nor for (**E**) culture-positive or (**F**) culture-negative site of infection. Boxes represent medians and inter quartile ranges, whiskers represent range. Individual values are overlayed on the plots. P values * *p* < 0.05, ** *p* < 0.01, were determined by one-way ANOVA and Mann Whitney test.

There was no significant difference in IgM levels between culture-positive and culture-negative patients (Figure 2A).

### 3.3. Comparison of Plasma Immunoglobulin M (IgM) Levels in Sepsis According to Culture-Negative or Positive Status

When comparing all culture-positive (combined Gram-positive and negative, CP, *n* = 28) to culture-negative (CN) samples (*n* = 16), with a calculated prevalence of 60% and 34%, respectively, no significant difference in IgM was observed (188 μg/mL (119–279) versus 166 μg/mL (114–297, *p* = 0.78) (Figure 2B). We then compared the IgM levels of culture-negative sepsis patients to SIRS patients (*p* = 0.025) and identified a significant difference particularly between SIRS patients and culture-negative sepsis survivors (*p* = 0.0055) (Figure 2C).

### 3.4. Correlation between Clinical Parameters and IgM Levels

We correlated relevant and available clinical parameters with IgM levels using Spearman correlation. IgM levels did not correlate with albumin (r = 0.058, *p* = 0.73), or immune markers TNFα (r = −0.027, *p* = 0.86), IL-1 (r = 0.1292, *p* = 0.91), IL-6 (r = −0.1615, *p* = 0.30), or IL-8 (r = −0.0209, *p* = 0.89) in the sepsis cohort. Comparing culture-positive versus negative sepsis, Table 3 summarizes significant (*p* < 0.05) correlations between plasma IgM levels. 

### 3.5. Diagnostic Evaluation of IgM Levels in Culture-Positive and Negative Samples

**Figure 3:**
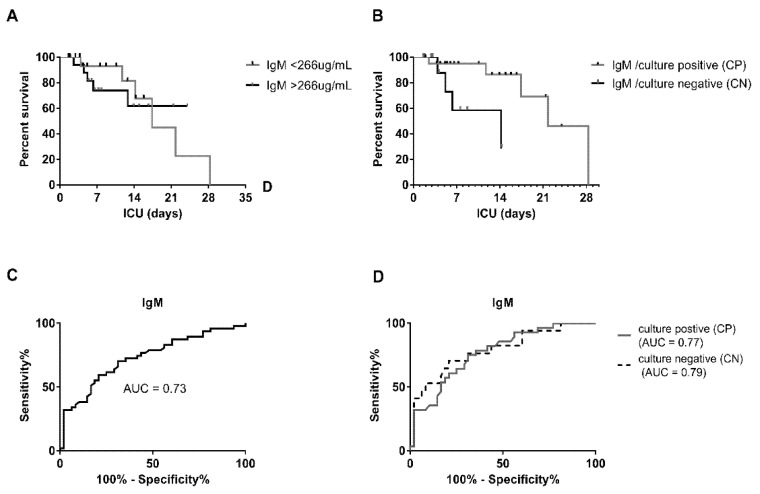
Survival and discrimination ability based on IgM levels in sepsis. (**A**) Kaplan–Meier survival curve based on IgM levels; (**B**) Kaplan–Meier survival curve based on culture positive or negative status. (**C**,**D**) Receiver operating characteristic curve for IgM levels comparing sepsis with healthy controls. AUC: area under the curve.

We used Kaplan–Meier curves to determine the survival in sepsis patients over ICU stay (days) for IgM levels < or > 266 μg/mL cut-off. Median survival was 17.4 days for IgM <266 μg/mL and for IgM > 266 μg/mL survival exceeded 50% at the longest time point; therefore, median survival could not be defined (Figure 3A). We found no significant statistical difference (*p* = 0.8607); however, a trend was observed until about day 14, which then disappeared due to cross-over of the survival curves. Number at risk was determined for IgM < 266 ug/mL at t(days): t0(18), t7(11), t14(6), t21(2), t28(1), and for IgM > 266 ug/mL at t(days): t0(18), t7(9), t14(5), t21(2), and t28(1). Comparing survival curves of IgM levels for CP with CN patients, we identified significantly increased survival in the CP patients with a median survival of 21.8 days, compared to 14.2 days for CN patients (*p* = 0.02) with a median survival ratio of 1.535 (95% CI of ratio: 0.4122 to 5.717). The corresponding number at risk for CP patients was t(days): t0(21), t7(13), t14(9), t21(4), t28(1), and for CN t(days): t0(12), t7(5), t14(5), t21(1), and t28(0) (Figure 3B). Receiver operating characteristic (ROC) curves were plotted comparing IgM in sepsis with healthy controls and the respective areas under the curve (AUC) was AUC = 0.73 (95% confidence interval (CI): 0.6278 to 0.8314, *p* = 0.0001) with a 70% sensitivity and 69% specificity at cut-off <266 μg/mL and a positive predictive value (PPV) of 7.9% and negative predictive value (NPV) of 92.4% calculated for an estimated average incidence of 780/100,000 (Figure 3C). AUC for distinct culture-positive and negative patients was AUC = 0.77 (CP, 95% confidence interval (CI): 0.6647 to 0.8777, *p* < 0.0001; PPV 5.7%, NPV 97.1%, 288 μg/mL cut-off) and AUC = 0.79 (CN, 95% confidence interval (CI): 0.6558 to 0.9189, *p* = 0.0005; PPV 2.3%, NPV96.4%, 232 μg/mL cut-off), respectively (Figure 3D).

## 4. Discussion

The aim of this pilot study in a small cohort of patients with sepsis was to evaluate the association between IgM plasma levels and survival in sepsis and SIRS, and to explore the feasibility of this analysis in larger cohorts. Our study assessed IgM levels in sepsis and SIRS patients and showed no correlation between the main pro-inflammatory cytokines TNFα, IL-1, IL-6, and IL-8 with IgM. IgM levels were lower in the sepsis group when compared to SIRS and healthy controls. We could not find any difference in IgM levels between culture-positive and culture-negative sepsis patients. However, we have observed higher IgM levels in culture-negative non-survivors, and a significant difference between culture-negative sepsis patients and SIRS.. Our results should be put into context of several similar sized observational studies.

We found, that decreased plasma IgM is likely independent of the cytokine storm seen in sepsis. Further, no correlation between IgM and albumin suggests that hemodilution is unlikely the cause of lowered IgM we see in our sepsis cohort, which has also been observed by Bermejo-Martin et al. [27]. Clinical studies have found that the immune response in sepsis plays an important role [4]. A delicate balance between the hosts inflammatory and anti-inflammatory response is essential for sepsis recovery [4,39]. It has been shown that increased pro-inflammatory cytokine levels such as TNFα and IL-6 levels correlate with poor outcome in sepsis; however, their routine clinical utility remains questionable [4,40]. It has recently become much more apparent that sepsis mortality is not only linked to an uncontrolled pro-inflammatory response, but to immunosuppression [4]. The classic sepsis immunology postulated that the cytokine-mediated hyper-inflammatory phase is followed by a subsequent immune-suppressive phase [41,42]. However, recent investigations including ours on the same patient population revealed that immunosuppression is also apparent in the acute phase of sepsis and it could be reversed by targeting the effector cells of the human immune system [4,30,43]. We observed significantly lower IgM levels in our sepsis patients confirming findings of other authors and this could reflect the extent of immunosuppression in this cohort [14,15,27,29].

Our findings of low IgM in sepsis versus SIRS supports the results of previous studies, including Tamayo et al. [15], who reported a protective role of IgG1, IgM and IgA against mortality in postsurgical patients with septic shock. Venet et al. [14] showed a significant reduction in IgM in septic shock patients at day 1, which recovered within normal range at day 5. Giamrellos-Bourboulis et al. [28] also reported decreased IgM in patients with septic shock compared to those with systemic inflammatory response syndrome or severe sepsis. Bermejo-Martin et al. [44] showed that the combined presence of low levels of the endogenous immunoglobulins IgG1, IgM and IgA in plasma is associated with reduced survival in patients with severe sepsis or septic shock. A recent meta-analysis summarizing these findings showed that reduced IgM are associated with decreased sepsis survival [45]. All of our patients had multi-organ failure, with significant cardiovascular impairment as evidenced by the SOFA scores. Nonetheless, most studies did not analyze the potential influence of immunoglobulin levels in further stratified populations of Gram-positive or negative sepsis and culture-positive (CP) versus negative (CN) sepsis, which is documented inconsistently. We showed that in culture-positive (combined Gram-positive and negative, CP) compared to culture-negative (CN) samples, no difference in IgM was observed. While a recent systematic review and meta-analysis found no association of mortality with CP or CN [46], we observed that IgM levels differed in CN survivors versus non-survivors in our cohort, but did not reach statistical signficance. Non-survivors had significantly raised IgM levels, similar to SIRS (survivors) and the Kaplan–Meier analysis revealed the association of increased mortality in CN patients. These data indicate that there may be a difference in the pathophysiology of CP versus CN sepsis linked to mortality; and also between CN sepsis and SIRS. However, there are no mechanistic studies to date explaining IgM kinetics in sepsis. It becomes apparent that low IgM cannot necessarily be attributed to generic sepsis mortality, but is influenced by factors such as the presence of an infecting organism. It is difficult to characterize the relative contributions of altered Ig production, impaired endothelial recycling, and Ig consumption for opsonization in sepsis, due to the lack of a well-characterized regulatory loop for Ig homeostasis [47]. A very recent study on a similar patient population to ours suggested, that activation-associated B-cell death is partly responsible for the loss of IgM production in sepsis [31]. B-cell generated IgM is crucial for bacterial clearance [22], while lymphocyte exhaustion is a mechanism of immunosuppression in sepsis [30]. Low B cells levels may partly explain low IgM levels overall, but do not distinguish between the differences we have seen between CN and SIRS patients.

Secreted neutralizing antibodies are of central importance to the immune protection of the host, acting as a first line of defense. A second line of defense are pathogen-experienced memory B cells that are rapidly reactivated to produce antibodies (reactive humoral memory) [48]. There are two distinct types of memory B cells, including IgM memory B cells [21]. The lack of IgM memory B cells, independently from the cause of depletion, is associated with increased susceptibility to encapsulated bacterial infection [49]. Recently, a study found that lymphopenia present in 74.2% of sepsis patients was a result of selective depletion of memory B cells, with greater apoptotic depletion of class-switched and IgM memory cells [31]. This would result in a less efficient response to re-infection.

IgM alone may not be a rigorous predictor of survival on its own and should be tested in conjunction with other biomarkers, as was suggested previously [27]. Overall, as a diagnostic test, IgM has equivalent or better specificity and sensitivity for sepsis with and AUC of 0.73 compared with CRP with varying AUC of 0.6–0.9 reported for severe sepsis [37,50,51,52]. AUC for IgM in CN sepsis (AUC 0.79) was similar to the AUC in CP (AUC 0.77) sepsis patients. Consistent with recently published data, we found that low IgM levels were common in sepsis on day 1 [29]. These findings increase the external validity of our results.

In conjunction, we believe that IgM level quantification has the potential to distinguish patients with suspected sepsis as probable SIRS patients within a CN cohort (false-positives) and emphasizes the need for more sensitive tests for occlusive organisms potentially undetected in the CN patients (false-negatives). Indeed, a recent study reported that a third of 55% initially identified as CN severe sepsis was subsequently found to have non-infectious mimics [53]. Further, atypical infections were found in 8% of the patients in the CN patients [53]. Nevertheless, the persistent incidence of CN sepsis in multiple studies emphasizes the need to better understand the relationship of immunoglobulins, and particularly IgM, in CP versus CN patients [9,54,55,56,57,58]. Importantly, a similarly sized, single center study, compared to ours, found about 40% of cases of severe sepsis to be CN, whilst 36% of our patients never had a causative organism identified [10]. These findings also increase the external validity of our results.

CRP was significantly reduced in CN patients, but higher than in SIRS patients. CRP did correlate with IgM levels in the CP, but not in the CN patients. CRP is a well-known and widely used marker for infection, despite its recognized drawbacks for specificity and sensitivity [59]. It is the standard, readily available test in our center, used in daily clinical practice. Whilst procalcitonin and other inflammatory cytokines have been proposed to be better discriminators for sepsis, it is very likely that a multiplexed approach using traditional and novel biomarkers could be more successful [60]. To our knowledge, no other studies have looked at correlation of CRP and IgM levels in adult sepsis. Our data indicate that there may be a functional difference between CP and CN sepsis and SIRS patients, associated with different IgM levels.

There are some major limitations of the current study. First the number of patients recruited is low, as the study was designed as an exploratory pilot. Therefore, our results should be viewed with caution and would need to be confirmed in larger datasets, recruiting patients preferably using the new Sepsis-3 clinical criteria, divided into CP and CN groups. Although our inclusion criteria were based on the Sepsis-1 definitions, all of our patients fulfilled the Sepsis-3 criteria as well, in fact they all exhibited signs of multi-organ failure based on their SOFA scores. Second, we had low numbers of SIRS samples. This was a result of recruitment difficulties due to our stringent exclusion criteria. Other studies used out-of-hospital cardiac arrest as a model of SIRS, however recent data indicates that these patients indeed manifest a “sepsis-like” immunological response [61]. Another limitation is the observed trend towards a high female to male ratio in both the SIRS and the CN cohort. This is particularly relevant when comparing SIRS to CN sepsis patients, where we have seen the most significant differences in IgM levels. It has been reported that IgM levels are higher in females than in males [62,63,64], however, we did observe a significant difference in IgM between the high female frequency SIRS and CN cohort, concluding this effect to be gender independent. There was also a significant age difference between the SIRS and sepsis groups. Severe organ dysfunction of non-infectious origin such as acute severe asthma, major trauma, drug overdose and burns usually affects a different age-group as opposed to sepsis, and this is reflected in our results [65]. Due to a lack of data from previous studies, it is impossible to ascertain if this difference played any role in our findings. However, IgM levels have been found to be age and sex dependent in healthy individuals [63]. Whilst the SOFA scores were also lower in the SIRS group, this statistically significant difference is clinically irrelevant: all patients in both groups had multi-organ failure according to the original definition [38].

## 5. Conclusions

There is a clear lack of useful cohort stratification in sepsis pathology. Our findings support the idea that the stratification into CP and CN sepsis patients, in addition to SIRS, can be a useful tool to dissect distinct mechanisms. Identification of biomarkers such as IgM that potentially distinguish a mechanism will be instrumental to further elucidate sepsis pathology and to stratify immunoglobulin therapy by targeting likely responders.

We propose IgM and its role in early microbial clearance as a suitable biomarker that can aid the distinction of sepsis and SIRS and potentially identify SIRS patients within the culture-negative cohort.

Given the small sample size of our study, a larger study is required to confirm these pilot data, in particular the role of IgM in culture-negative sepsis pathology.

## Figures and Tables

**Table 1:** Comparison of patient characteristic, outcome, plasma biomarker levels and site of infection in sepsis and SIRS.

	Sepsis *n* = 47	SIRS *n* = 11	*p* Value
Age	63 (54–71)	42 (34–65)	0.03
Gender (male/female)	23/24	2/9	-
ICU days	8 (4–17)	6 (3–22)	0.59
Hospital days	27 (15–66)	20 (15–26)	0.25
ICU mortality	16/47 (34%)	1/11 (9%)	-
APACHE II score	17 (14–21)	12 (9–21)	0.29
SOFA Day 1	15 (13–17)	14 (3–15)	0.02
SOFA Day 5	13 (9–15)	8 (6–12)	0.01
Plasma markers			
IgM (μg/mL)	193 (120–310)	331 (236–581)	0.03
IgM (survivor)	181 (117–298)	370 (243–650)	0.002
IgM (non-survivor)	242 (154–350)	92 (*n* = 1)	-
CRP (μg/mL)	213 (130–300)	28 (9–72)	0.0001
Site of Infection			
Abdominal	17 (40%)	-	-
Respiratory	15 (36%)	-	-
Urinary tract	5 (12%)	-	-
Soft tissue	5 (12%)	-	-

Data is presented as frequency (%) or median and interquartile range, (IQR) as appropriate. An unpaired non-parametric Mann–Whitney U test was used to determine the *p* value. ICU: Intensive care unit; APACHE II: Acute Physiology and Chronic Health Evaluation Score II; SOFA: Sequential Organ Failure Assessment score; IgM: Immunoglobulin M; CRP: C-reactive protein.

**Table 2:** Comparison of severe sepsis patient characteristic, outcome, plasma biomarker levels and site of infection.

	Culture-Positive (CP) *n* = 28	Culture-Negative (CN) *n* = 16	*p* Value
Age	62 (54–69)	65 (49–76)	0.68
Gender (male/female)	16/11	4/10	-
ICU days	13 (5–22)	6 (3–10)	0.02
Hospital days	31 (15–66)	22 (13–42)	0.53
ICU outcome	26% mortality	29% mortality	0.99
APACHE II score	19 (15–21)	16 (13–23)	0.56
SOFA Day 1	15 (13–17)	14 (12–17)	0.57
SOFA Day 5	12 (9–15)	13 (12–14)	0.60
Plasma markers			
IgM (μg/mL)	188 (119–279)	166 (114–297)	0.78
IgM (survivors)	222 (118–312)	124 (112–189)	0.14
IgM (non-survivors)	181 (129–240)	310 (151–364)	0.18
CRP (μg/mL)	231 (143–304)	145 (23–280)	0.07
Site of Infection			
Abdominal	12 (42%)	5 (36%)	0.98
Respiratory	10 (36%)	5 (36%)	0.76
Urinary tract	3 (11%)	2 (14%)	0.20
Soft tissue	3 (11%)	2 (14%)	0.40

Data is presented as frequency (%) or median and interquartile range, (IQR) as appropriate. An unpaired non-parametric Mann–Whitney U test was used to determine the *p* value. ICU: Intensive care unit; APACHE II: Acute Physiology and Chronic Health Evaluation Score II; SOFA: Sequential Organ Failure Assessment score; IgM: Immunoglobulin M; CRP: C-reactive protein.

**Table 3:** Spearman non-parametric correlation (r) of IgM levels with SOFA scores and plasma biomarkers in culture-positive and negative sepsis patients.

IgM Correlation	Culture-Positive (CP)	*p* Value	Culture-Negative (CN)	*p* Value
SOFA day 1	r = −0.23 (*n* = 22)	0.29	r = −0.608 (*n* = 11)	0.04
SOFA day 5	r = 0.17 (*n* = 23)	0.41	r = −0.707 (*n* = 10)	0.01
CRP	r = −0.52 (*n* = 27)	0.004	r = 0.23 (*n* = 16)	0.38

SOFA: Sequential Organ Failure Assessment score; IgM: Immunoglobulin M; CRP: C-reactive protein. For statistical analysis Spearman’s Rho test was used. We observed a statistically significant correlation of IgM levels with SOFA score in the CN (d1, *p* = 0.04; d5 *p* = 0.01), but not CP patients. IgM levels correlated with CRP in the CP patients (*p* = 0.004), but not in the CN cohort.

## Data Availability

The data presented in this study are available on request from the corresponding author, Meike Heurich, School of Pharmacy and Pharmaceutical Sciences, Cardiff University, Cardiff, UK; e-mail: heurichm@cardiff.ac.uk. The data are not publicly available due to privacy and ethical restrictions.

## References

[1] Singer M., Deutschman C.S., Seymour C.W., Shankar-Hari M., Annane D., Bauer M., Bellomo R., Bernard G.R., Chiche J.-D., Coopersmith C.M. (2016). The third international consensus definitions for sepsis and septic shock (sepsis-3). JAMA.

[2] Bone R.C., Balk R.A., Cerra F.B., Dellinger R.P., Fein A.M., Knaus W.A., Schein R.M., Sibbald W.J. (1992). Definitions for sepsis and organ failure and guidelines for the use of innovative therapies in sepsis. The ACCP/SCCM Consensus Conference Committee. American College of Chest Physicians/Society of Critical Care Medicine. Chest.

[3] Kaukonen K.M., Bailey M., Suzuki S., Pilcher D., Bellomo R. (2014). Mortality related to severe sepsis and septic shock among critically ill patients in Australia and New Zealand, 2000–2012. JAMA.

[4] Hotchkiss R.S., Moldawer L.L., Opal S.M., Reinhart K., Turnbull I.R., Vincent J.-L. (2016). Sepsis and septic shock. Nat. Rev. Dis. Primers.

[5] Ramachandran G. (2014). Gram-positive and gram-negative bacterial toxins in sepsis: A brief review. Virulence.

[6] Sriskandan S., Cohen J. (1999). Gram-positive sepsis. Mechanisms and differences from gram-negative sepsis. Infect. Dis. Clin. N. Am..

[7] Dellinger R.P., Levy M.M., Rhodes A., Annane D., Gerlach H., Opal S.M., Sevransky J.E., Sprung C.L., Douglas I.S., Jaeschke R. (2013). Surviving sepsis campaign: International guidelines for management of severe sepsis and septic shock: 2012. Crit. Care Med..

[8] Morgan M.P., Szakmany T., Power S.G., Olaniyi P., Hall J.E., Rowan K., Eberl M. (2016). Sepsis Patients with First and Second-Hit Infections Show Different Outcomes Depending on the Causative Organism. Front. Microbiol..

[9] Martin G.S., Mannino D.M., Eaton S., Moss M. (2003). The epidemiology of sepsis in the United States from 1979 through 2000. N. Engl. J. Med..

[10] Gupta S., Sakhuja A., Kumar G., McGrath E., Nanchal R.S., Kashani K.B. (2016). Culture-Negative Severe Sepsis: Nationwide Trends and Outcomes. Chest.

[11] Phua J., Ngerng W.J., See K.C., Tay C.K., Kiong T., Lim H.F., Chew M.Y., Yip H.S., Tan A., Khalizah H.J. (2013). Characteristics and outcomes of culture-negative versus culture-positive severe sepsis. Crit. Care.

[12] Cohen J., Vincent J.L., Adhikari N.K., Machado F.R., Angus D.C., Calandra T., Jaton K., Giulieri S., Delaloye J., Opal S. (2015). Sepsis: A roadmap for future research. Lancet Infect. Dis..

[13] Rangel-Frausto M.S., Pittet D., Costigan M., Hwang T., Davis C.S., Wenzel R.P. (1995). The natural history of the systemic inflammatory response syndrome (SIRS). A prospective study. JAMA.

[14] Venet F., Gebeile R., Bancel J., Guignant C., Poitevin-Later F., Malcus C., Lepape A., Monneret G. (2011). Assessment of plasmatic immunoglobulin G, A and M levels in septic shock patients. Int. Immunopharmacol..

[15] Tamayo E., Fernández A., Almansa R., Carrasco E., Goncalves L., Heredia M., Andaluz-Ojeda D., March G., Rico L., Gómez-Herreras J.I. (2012). Beneficial role of endogenous immunoglobulin subclasses and isotypes in septic shock. J. Crit. Care.

[16] Prucha M., Zazula R., Herold I., Dostal M., Hyanek T., Bellingan G. (2014). Presence of hypogammaglobulinemia in patients with severe sepsis, septic shock, and SIRS is associated with increased mortality. J. Infect..

[17] Taccone F.S., Stordeur P., De Backer D., Creteur J., Vincent J.L. (2009). Gamma-globulin levels in patients with community-acquired septic shock. Shock.

[18] Dietz S., Lautenschlaeger C., Mueller-Werdan U., Werdan K. (2010). Low levels of immunoglobulin G in patients with sepsis or septic shock: A signum mali ominis?. Crit. Care.

[19] Racine R., Winslow G.M. (2009). IgM in microbial infections: Taken for granted?. Immunol. Lett..

[20] Yates J.L., Racine R., McBride K.M., Winslow G.M. (2013). T cell-dependent IgM Memory B Cells Generated During Bacterial Infection are Required for IgG Responses to Antigen Challenge. J. Immunol..

[21] Kurosaki T., Kometani K., Ise W. (2015). Memory B cells. Nat. Rev. Immunol..

[22] Boes M., Prodeus A.P., Schmidt T., Carroll M.C., Chen J. (1998). A critical role of natural immunoglobulin M in immediate defense against systemic bacterial infection. J. Exp. Med..

[23] Baumgarth N., Herman O.C., Jager G.C., Brown L.E., Herzenberg L.A., Chen J. (2000). B-1 and B-2 cell-derived immunoglobulin M antibodies are nonredundant components of the protective response to influenza virus infection. J. Exp. Med..

[24] Ochsenbein A.F., Fehr T., Lutz C., Suter M., Brombacher F., Hengartner H., Zinkernagel R.M. (1999). Control of early viral and bacterial distribution and disease by natural antibodies. Science.

[25] Boes M., Esau C., Fischer M.B., Schmidt T., Carroll M., Chen J. (1998). Enhanced B-1 cell development, but impaired IgG antibody responses in mice deficient in secreted IgM. J. Immunol..

[26] Gupta S., Gupta A. (2017). Selective IgM Deficiency—An Underestimated Primary Immunodeficiency. Front. Immunol..

[27] Bermejo-Martín J.F., Rodriguez-Fernandez A., Herrán-Monge R., Andaluz-Ojeda D., Muriel-Bombín A., Merino P., García-García M.M., Citores R., Gandía F., Almansa R. (2014). Immunoglobulins IgG1, IgM and IgA: A synergistic team influencing survival in sepsis. J. Intern. Med..

[28] Giamarellos-Bourboulis E.J., Apostolidou E., Lada M., Perdios I., Gatselis N.K., Tsangaris I., Georgitsi M., Bristianou M., Kanni T., Sereti K. (2013). Kinetics of circulating immunoglobulin M in sepsis: Relationship with final outcome. Crit. Care.

[29] Shankar-Hari M., Singer M., Spencer J. (2017). Can Concurrent Abnormalities in Free Light Chains and Immunoglobulin Concentrations Identify a Target Population for Immunoglobulin Trials in Sepsis?. Crit. Care Med..

[30] Boomer J.S., To K., Chang K.C., Takasu O., Osborne D.F., Walton A.H., Bricker T.L., Jarman S.D., Kreisel D., Krupnick A.S. (2011). Immunosuppression in patients who die of sepsis and multiple organ failure. JAMA.

[31] Shankar-Hari M., Fear D., Lavender P., Mare T., Beale R., Swanson C., Singer M., Spencer J. (2017). Activation-Associated Accelerated Apoptosis of Memory B Cells in Critically Ill Patients with Sepsis. Crit. Care Med..

[32] Tagami T., Matsui H., Fushimi K., Yasunaga H. (2015). Intravenous immunoglobulin use in septic shock patients after emergency laparotomy. J. Infect..

[33] Busani S., Damiani E., Cavazzuti I., Donati A., Girardis M. (2016). Intravenous immunoglobulin in septic shock: Review of the mechanisms of action and meta-analysis of the clinical effectiveness. Minerva Anestesiol..

[34] Toth I., Mikor A., Leiner T., Molnar Z., Bogar L., Szakmany T. (2013). Effects of IgM-enriched immunoglobulin therapy in septic-shock-induced multiple organ failure: Pilot study. J. Anesth..

[35] Szakmany T., Heurich-Sevcenco M. (2015). Immunomodulation in sepsis-why blunting the response doesn’t work?. J. Infect..

[36] Holst B., Szakmany T., Raby A.-C., Hamlyn V., Durno K., Hall J.E., Labéta M.O. (2017). Soluble Toll-like receptor 2 is a biomarker for sepsis in critically ill patients with multi-organ failure within 12 h of ICU admission. Intensive Care Med. Exp..

[37] Jones A.E., Trzeciak S., Kline J.A. (2009). The Sequential Organ Failure Assessment score for predicting outcome in patients with severe sepsis and evidence of hypoperfusion at the time of emergency department presentation. Crit. Care Med..

[38] Vincent J.L., Moreno R., Takala J., Willatts S., De Mendonça A., Bruining H., Reinhart C.K., Suter P.M., Thijs L.G. (1996). The SOFA (Sepsis-related Organ Failure Assessment) score to describe organ dysfunction/failure. On behalf of the Working Group on Sepsis-Related Problems of the European Society of Intensive Care Medicine. Intensive Care Med..

[39] Wiersinga W.J., Leopold S.J., Cranendonk D.R., van der Poll T. (2014). Host innate immune responses to sepsis. Virulence.

[40] Pinsky M.R., Vincent J.-L., Deviere J., Alegre M., Kahn R.J., Dupont E. (1993). Serum Cytokine Levels in Human Septic Shock: Relation to Multiple-System Organ Failure and Mortality. Chest.

[41] Chen X.-H., Yin Y.-J., Zhang J.-X. (2011). Sepsis and immune response. World J. Emerg. Med..

[42] Boomer J.S., Green J.M., Hotchkiss R.S. (2014). The changing immune system in sepsis: Is individualized immuno-modulatory therapy the answer?. Virulence.

[43] Raby A.C., Holst B., Le Bouder E., Diaz C., Ferran E., Conraux L., Guillemot J.C., Coles B., Kift-Morgan A., Colmont C.S. (2013). Targeting the TLR co-receptor CD14 with TLR2-derived peptides modulates immune responses to pathogens. Sci. Transl. Med..

[44] Bermejo-Martin J.F., Giamarellos-Bourboulis E.J. (2015). Endogenous immunoglobulins and sepsis: New perspectives for guiding replacement therapies. Int. J. Antimicrob. Agents.

[45] Krautz C., Maier S.L., Brunner M., Langheinrich M., Giamarellos-Bourboulis E.J., Gogos C., Armaganidis A., Kunath F., Grützmann R., Weber G.F. (2018). Reduced circulating B cells and plasma IgM levels are associated with decreased survival in sepsis—A meta-analysis. J. Crit. Care.

[46] Li Y., Guo J., Yang H., Li H., Shen Y., Zhang D. (2021). Comparison of culture-negative and culture-positive sepsis or septic shock: A systematic review and meta-analysis. Crit. Care.

[47] Junghans R.P. (1997). IgG Biosynthesis: No “Immunoregulatory Feedback”. Blood.

[48] Ahmed R., Gray D. (1996). Immunological memory and protective immunity: Understanding their relation. Science.

[49] Capolunghi F., Rosado M.M., Sinibaldi M., Aranburu A., Carsetti R. (2013). Why do we need IgM memory B cells?. Immunol. Lett..

[50] Uusitalo-Seppala R., Huttunen R., Aittoniemi J., Koskinen P., Leino A., Vahlberg T., Rintala E.M. (2013). Pentraxin 3 (PTX3) is associated with severe sepsis and fatal disease in emergency room patients with suspected infection: A prospective cohort study. PLoS ONE.

[51] Devran Ö., Karakurt Z., Adıgüzel N., Güngör G., Moçin Ö.Y., Balcı M.K., Çelik E., Saltürk C., Takır H.B., Kargın F. (2012). C-reactive protein as a predictor of mortality in patients affected with severe sepsis in intensive care unit. Multidiscip. Respir. Med..

[52] Feng L., Zhou X., Su L.-X., Feng D., Jia Y.-H., Xie L.-X. (2012). Clinical Significance of Soluble Hemoglobin Scavenger Receptor CD163 (sCD163) in Sepsis, a Prospective Study. PLoS ONE.

[53] Heffner A.C., Horton J.M., Marchick M.R., Jones A.E. (2010). Etiology of illness in patients with severe sepsis admitted to the hospital from the emergency department. Clin. Infect. Dis..

[54] Kumar A., Roberts D., Wood K.E., Light B., Parrillo J.E., Sharma S., Suppes R., Feinstein D., Zanotti S., Taiberg L. (2006). Duration of hypotension before initiation of effective antimicrobial therapy is the critical determinant of survival in human septic shock. Crit. Care Med..

[55] Vincent J.L., Sakr Y., Sprung C.L., Ranieri V.M., Reinhart K., Gerlach H., Moreno R., Carlet J., Le Gall J.R., Payen D. (2006). Sepsis in European intensive care units: Results of the SOAP study. Crit. Care Med..

[56] Blanco J., Muriel-Bombin A., Sagredo V., Taboada F., Gandia F., Tamayo L., Collado J., Garcia-Labattut A., Carriedo D., Valledor M. (2008). Incidence, organ dysfunction and mortality in severe sepsis: A Spanish multicentre study. Crit. Care.

[57] Brun-Buisson C., Meshaka P., Pinton P., Vallet B. (2004). EPISEPSIS: A reappraisal of the epidemiology and outcome of severe sepsis in French intensive care units. Intensive Care Med.

[58] Martin C.M., Priestap F., Fisher H., Fowler R.A., Heyland D.K., Keenan S.P., Longo C.J., Morrison T., Bentley D., Antman N. (2009). A prospective, observational registry of patients with severe sepsis: The Canadian Sepsis Treatment and Response Registry. Crit. Care Med..

[59] Wu C.C., Lan H.M., Han S.T., Chaou C.H., Yeh C.F., Liu S.H., Li C.H., Blaney G.N., Liu Z.Y., Chen K.F. (2017). Comparison of diagnostic accuracy in sepsis between presepsin, procalcitonin, and C-reactive protein: A systematic review and meta-analysis. Ann. Intensive Care.

[60] Kempsell K.E., Ball G., Szakmany T. (2016). Issues in biomarker identification, validation and development for disease diagnostics in Public Health. Expert Rev. Mol. Diagn..

[61] Adrie C., Adib-Conquy M., Laurent I., Monchi M., Vinsonneau C., Fitting C., Fraisse F., Dinh-Xuan A.T., Carli P., Spaulding C. (2002). Successful cardiopulmonary resuscitation after cardiac arrest as a “sepsis-like” syndrome. Circulation.

[62] Maddison S.E., Reimer C.B. (1976). Normative values of serum immunoglobulins by single radial immunodiffusion: A review. Clin. Chem..

[63] Stoica G., Macarie E., Michiu V., Stoica R.C. (1980). Biologic variation of human immunoglobulin concentration. I. Sex-age specific effects on serum levels of IgG, IgA, IgM and IgD. Med. Interne.

[64] Giltay E.J., Fonk J.C., von Blomberg B.M., Drexhage H.A., Schalkwijk C., Gooren L.J. (2000). In vivo effects of sex steroids on lymphocyte responsiveness and immunoglobulin levels in humans. J. Clin. Endocrinol. Metab..

[65] Brun-Buisson C. (2000). The epidemiology of the systemic inflammatory response. Intensive Care Med.

